# Transmissibility versus Pathogenicity of Self-Propagating Protein Aggregates

**DOI:** 10.3390/v11111044

**Published:** 2019-11-09

**Authors:** Byron Caughey, Allison Kraus

**Affiliations:** 1 Laboratory of Persistent Viral Diseases, Rocky Mountain Laboratories, National Institute of Allergy and Infectious Diseases, National Institutes of Health, Hamilton, MT 59840, USA; 2 Department of Pathology, Case Western Reserve University School of Medicine, Cleveland, OH 44106, USA

**Keywords:** prion, amyloid, infectivity, pathogenesis, transmission, tau, synuclein, amyloid-β

## Abstract

The prion-like spreading and accumulation of specific protein aggregates appear to be central to the pathogenesis of many human diseases, including Alzheimer’s and Parkinson’s. Accumulating evidence indicates that inoculation of tissue extracts from diseased individuals into suitable experimental animals can in many cases induce the aggregation of the disease-associated protein, as well as related pathological lesions. These findings, together with the history of the prion field, have raised the questions about whether such disease-associated protein aggregates are transmissible between humans by casual or iatrogenic routes, and, if so, do they propagate enough in the new host to cause disease? These practical considerations are important because real, and perhaps even only imagined, risks of human-to-human transmission of diseases such as Alzheimer’s and Parkinson’s may force costly changes in clinical practice that, in turn, are likely to have unintended consequences. The prion field has taught us that a single protein, PrP, can aggregate into forms that can propagate exponentially in vitro, but range from being innocuous to deadly when injected into experimental animals in ways that depend strongly on factors such as conformational subtleties, routes of inoculation, and host responses. In assessing the hazards posed by various disease-associated, self-propagating protein aggregates, it is imperative to consider both their actual transmissibilities and the pathological consequences of their propagation, if any, in recipient hosts.

## 1. Introduction

Even before the term “prion” was coined, the mysterious infectious agents of scrapie in sheep and goats, and kuru of the then-endocannibalistic Fore-speaking people of the highlands of New Guinea captured considerable attention. The remarkable resistance of these agents to environmental degradation, radiation, and chemical disinfectants elicited prescient arguments by J.S. Griffith that corrupted proteins could be pathogens via replication mechanisms involving aberrant conformational change akin to nucleated condensation [1]. Carleton Gajdusek won the Nobel Prize in 1976 for his work showing that kuru was experimentally transmissible to non-human primates, and therefore likely to be transmitted among the Fore by ritualistic consumption of family members who had died of the disease (reviewed in [2]). Gajdusek’s transmission experiments were suggested to him by veterinary pathologist William Hadlow, who recognized similarities between the neuropathologies of kuru and scrapie, which by then was known to be transmissible [3]. Among the prominent neuropathological features of kuru were amyloid plaques, which were later shown to contain the protein (prion protein or PrP) identified by Stanley Prusiner and colleagues as the key molecular component of prion disease infectivity [4]. Gajdusek [5,6] and Peter Lansbury [7] described the fundamental self-propagating, and thereby potentially infectious, nature of protein amyloid fibrils via seeded polymerization mechanisms. Indeed, Gajdusek initially described kuru as an apparent example of “galloping senescence of the juvenile”, which seems to invoke the rampant amyloid-β deposits of Alzheimer’s disease. He would often expound on the abundant precedents in the natural world for seeded, pattern-setting growth of materials ranging from mineral crystals to amyloid fibrils. He, and more formally Peter Lansbury [8], likened the growth of ordered protein aggregates to Kurt Vonnegut’s fictional Ice-Nine. Ice-Nine rarely formed spontaneously due to the metastability of liquid water and the kinetic favorability of forming common ice upon cooling. However, once formed, the more stable Ice-Nine grew uncontrollably, irreversibly, and catastrophically through seeded crystallization of all the liquid water it touched.

In many ways, protein amyloids can be seen as one-dimensional crystals [7]. Many, if not most, proteins can assemble into amyloid fibrils under appropriate conditions [9], which in many cases can be physiological and pathological [9,10]. Indeed, the list of polypeptides that aggregate in the context of human diseases has grown very long [11]. Among the most prominent examples of these are Aβ (in Alzheimer’s disease), tau (e.g., in Alzheimer’s, chronic traumatic encephalopathy, and progressive supranuclear palsy), α-synuclein (e.g., in Parkinson’s disease, dementia with Lewy bodies, and multiple system atrophy), TDP-43 (in amyotrophic lateral sclerosis and frontotemporal dementia), and huntingtin (in Huntington’s disease). Although some diseases are closely linked to pathological mutations in the genes of the aggregating proteins, many others involve accumulation of wild type proteins or fragments thereof. Multiple factors can influence the extent to which abnormal protein aggregation causes problems in the host including: The rate of aggregation; the rate of aggregate degradation by protein quality control mechanisms; the rates of aggregate propagation within and between cells and tissues; the efficacy of protective compensatory responses; and the types of inflammatory responses, which can either ameliorate or exacerbate pathogenesis ([12,13,14] and references therein). Most protein folding diseases increase in incidence after middle age, suggesting that there are age-dependent declines in our ability to control and/or tolerate protein aggregation.

## 2. Prion, and Prion-Like, Diseases

The most clearly documented way of overwhelming physiological defenses against protein aggregation is the injection of aggregates from diseased individuals. With experimental rodent models of PrP-based prion diseases (transmissible spongiform encephalopathies, or TSE), this process occurs like clockwork, with inoculation of a given dose of a given prion by a specific route causing fatal disease after a highly predictable incubation period months after inoculation. The term prion, whether applied to mammals or fungi, has traditionally referred to protein-based infectious agents or elements of inheritance that lack their own nucleic acid genome [15,16]. This concept has usually included the characteristic of transmissibility between individual organisms. However, there are now plentiful examples of self-propagating, often aggregated, states of various proteins that are well-documented to spread between cells and tissues of the host (reviewed in [17,18,19]), but without clear evidence of transmission between individuals, at least by practical or natural routes. Many scientists are describing such intermediate self-propagating protein states as “prion-like”, while others prefer to simply call them all prions [20,21].

## 3. Transmissibility versus Pathogenicity of PrP Aggregation

**Figure 1:**
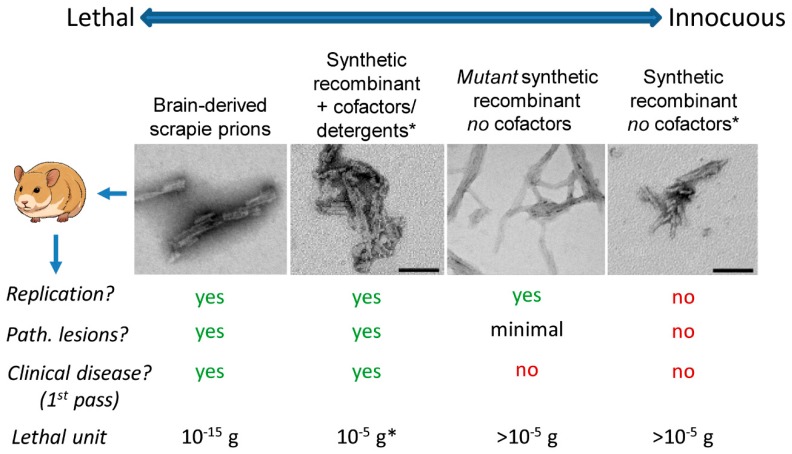
Spectrum of transmissibilities and pathogenicities of hamster PrP amyloids. Each of the pictured amyloid fibrils can efficiently seed the continuous propagation of amyloid fibrils in vitro, but differ markedly in the consequences of their inoculation in vivo. The images are negatively stained transmission electron micrographs with the bar spanning 100 nm. * These panels adapted from [31]. Although the fibrils depicted in second panel from left was prepared with detergent [31] and only barely lethal [32], it is important to note that other synthetic recombinant prions prepared with cofactors can be orders of magnitude more lethal per unit protein (lethal unit of ~10^−10^ g) [33]. “Path.” stands for neuropathological. For further information on the “mutant synthetic recombinant no cofactors” fibrils, see [34].

Nomenclature aside, it is important to understand that even a single protein can be assembled into a spectrum of self-propagating states (e.g., conformers or “strains”) that, if inoculated in vivo, can have starkly different consequences for the host. For example, Syrian hamster PrP molecules alone can assemble into multiple types of amyloid fibrils, each of which can be propagated indefinitely in vitro but, when inoculated into animals, can range from being lethal to totally innocuous (Figure 1). Here we use PrP to refer generically to the prion protein, which can exist in normal (PrP^C^), infectious and pathological (e.g., PrP-scrapie or PrP^Sc^), or intermediate states. On the lethal end of the spectrum of PrP aggregates are brain-derived preparations of PrP^Sc^, a single microgram of which could kill roughly a billion hamsters if suitably diluted and inoculated intracerebrally (e.g., [22]). On the other end are multiple examples of synthetic recombinant PrP fibrils that fail to propagate or cause neuropathology, even if micrograms are inoculated into a single animal (e.g., [23,24,25]). Wildtype brain-derived PrP^Sc^ may be most transmissible because of the presence of subamyloid [26], as well as amyloid, ultrastructures that have higher specific infectivity (per unit protein) than larger amyloid fibrils [27]. However, this does not mean that amyloid fibrils of PrP^Sc^ are not highly infectious and pathogenic, too, as has been strongly evidenced by multiple studies, e.g., [27,28,29,30].

Key studies describing the fundamental molecular composition of prions showed that pathogenic prions can be assembled in vitro using pure recombinant PrP molecules and cofactors such as polyanions, phospholipids, or detergents [32,35,36,37]. However, multiple other attempts to generate prions from defined molecular components in vitro have yielded synthetic PrP fibril preparations that, when inoculated into rodents, can initiate the accumulation of abnormal deposits of PrP or million-fold amplification of prion seeding activity, but without causing clinical disease within the lifespan of the host [23,24,38,39,40,41]. In some of these examples, abnormal PrP accumulation occurred without much, if any, neuropathology. Indeed, in one recent study, Diaz-Espinoza and colleagues showed that prophylactic injection of non-toxic, self-replicating PrP fibrils into hamsters was actually protective against subsequent inoculation with fully pathogenic scrapie prions [42]. These studies have provided clear examples of infectious (transmissible and self-propagating) but non-pathogenic aggregates of PrP. Interestingly, in several of these studies, second passage of brain tissue from such mice eventually caused clinical prion disease, a process that was typically accompanied by the accumulation of differently structured PrP^Sc^. Conformational adaptation or shifting of prions through the propagation process under selective conditions has been described as “Darwinian evolution of prions” [43] or “deformed templating” [44].

**Figure 2:**
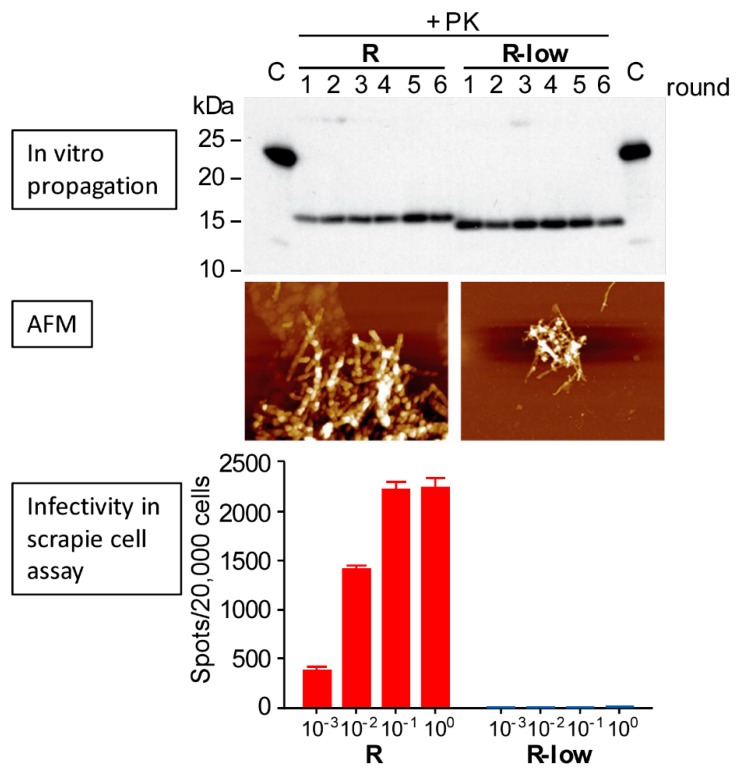
Seemingly subtle conformational differences between two conformers (R and R-low) of murine PrP fibrils formed and propagated under similar conditions in vitro can have dramatic differences in infectivity [24]. Both the “R “and “R-low” fibrils were generated in serial protein folding cyclic amplification reactions containing recombinant PrP, total mouse liver RNA, and a synthetic phospholipid. Top: The “R” and “R-low” fibrils maintain a ~1 kDa difference in their proteinase K (PK)-resistant cores through 6 rounds of amplification. Middle: Atomic force microscopy (AFM) images of the respective “R” (left) and “R-low” (right) preparations indicate that both have fibrillar ultrastructures. Bottom: Relative levels of infectivity measured on a live-cell based assay across four 10-fold dilutions, indicating a difference in infectivity of at least 10^4^. Adapted with permission from [24].

Thus, both pathogenic and non-pathogenic PrP aggregates can be generated in vitro, and conditions have been determined that lead to the reproducible in vitro generation of lethal PrP amyloids. Yet even seemingly subtle differences in biochemical or biophysical characteristics of PrP amyloids can lead to stark divergence in the resulting lethality in a host [45,46]. For example, a difference of only ~1 kDa in proteinase K-resistant amyloid cores (with respect to the mass of the constituent PrP monomers) can correlate with either lethal or clinically innocuous outcomes of inoculation into rodents [24] (Figure 2). The addition of cofactors to otherwise non-pathogenic PrP aggregates can also lead to a rapid and dramatic increase in infectivity and pathogenicity [25]. Substitution of specific lysine and proline residues in recombinant PrP molecules can also promote the generation of aggregates that, even without polyanionic or phospholipid cofactors, are capable of seeding further prion formation in the host, albeit without causing clinical disease on first passage [23,41]. These specific lysine residues have been suggested to be important in polyanionic cofactor binding [47,48,49].

## 4. Transmission of, and Susceptibility to, Human Prion Diseases

Of particular interest with respect to transmissibility versus pathogenicity are the diversity of outcomes when brain tissue from humans who died of various familial prion diseases have been inoculated into non-human primates or humanized mice (reviewed in [50,51]). Thirty-four different mutations in human prion protein have been associated with development of genetic human prion diseases. Of these, tissue derived from patients representing 13 of these PrP mutations thus far have been tested in transmission experiments into rodent or primate models, with only nine showing evidence of transmission (reviewed in [50,51]). While assessments of transmissibilities to human subjects are potentially limited by unknown constraints on the ability of specific human prions to propagate within a particular animal model, these results clearly indicate striking variability in the infectious and/or pathogenic properties of forms of human PrP that are pathological in the original human host. This may reflect differences in the mechanisms by which different PrP mutations result in PrP aggregate/prion formation. Certain PrP mutations may promote formation of transmissible, pathogenic prions [52], whereas others appear to destabilize the native prion protein to promote the formation of pathogenic but not necessarily transmissible PrP aggregates [51,53,54,55,56].

Transmission of prion disease may also depend on individual host responses, such that in a heterogeneous human population, some people may be more susceptible than others. For example, almost all genotyped cases of variant Creutzfeldt-Jakob disease, which were presumably due to consumption of prion-tainted beef, have been homozygous for methionine at polymorphic residue 129 of *PRNP* [57,58]. *PRNP* genotype also affects human susceptibility to kuru [59], and both iatrogenic and sporadic forms of Creutzfeldt-Jakob disease [60,61]. In addition, there are multiple examples of PRNP polymorphisms that influence prion disease susceptibility in other mammals (reviewed in [62]). Collectively, these findings underscore the importance of avoiding a ‘one-size fits all’ approach when assessing biological risks of self-propagating PrP aggregates.

## 5. Implications for Other Types of Proteopathies

Understanding pathogenic versus non-pathogenic distinctions between self-propagating amyloids or other protein aggregates has implications far beyond prions and PrP amyloids. For example, growing evidence suggests that aggregates of proteins such as Aβ, tau, and α-synuclein accumulate with predictable staging in progressively wider neuroanatomical areas in the course of Alzheimer’s and Parkinson’s diseases, respectively [63,64]. There are also multiple experimental demonstrations that inoculation of diseased tissues from patients with these and other protein folding diseases can initiate accumulation of analogous abnormal protein aggregates and related pathologies in experimental animals [17,18,65,66,67]. As these experimental transmissions are usually unnatural manipulations in unnatural hosts, and as such may not recapitulate plausible modes of human-to-human transmissions, it remains critical to evaluate the extent to which analogous transmissions might contribute to morbidity and mortality in humans.

Importantly, as with some of the PrP-based experimental scenarios described above, it seems relatively commonplace to see brain deposits of proteins such as Aβ, tau, and α-synuclein in people without clinically apparent brain disease [68,69]. For example, senile plaques of Aβ akin to those of Alzheimer’s disease (AD) are frequently found in elderly cognitively normal people. Alzheimer’s-like tau deposits also regularly occur in cognitively normal individuals, with particularly high prevalence in elderly populations. In fact, a relatively recently defined neuropathological diagnoses, primary age-related tauopathy (PART), describes the largely non-clinical age-related occurrence of AD-like (i.e., 3R/4R) tau deposits, primarily occurring in the temporal lobe [70]. The neuropathological identification of PART is defined by a Braak classification of IV or less with no or little Aβ deposition [70], which has raised questions in the field as to whether PART, given more time, may proceed to AD [71], or if it represents a separate occurrence of tau deposits in the brain [72,73]. The clinicopathological spectrum of PART is not yet fully characterized, but in some cases, PART with higher Braak stages (III/IV) can be associated with cognitive impairment and increased neuropathological changes [70,72]. It remains unclear if the tau aggregates that define PART represent a self-propagating tau conformer, but recent studies suggest that brain tissue derived from PART cases can at least seed further amyloid formation in cell-based tau seeding assays [74] and real-time quaking induced conversion (RT-QuIC) seed amplification assays [75]. Yet, the often sub-clinical outcomes of PART neuropathologies may suggest a limitation of the PART-related tau aggregates to amplify in vivo, or to amplify in ways that readily elicit clinical outcomes.

A number of studies have indicated that misfolded tau and α-synuclein can occur as different structural assemblies that behave like prion strains, with different seeding capacities, clinical pathologies, and neurotoxic phenotypes in rodent and cellular models e.g., [65,76,77,78,79,80,81]. In fact, akin to PrP amyloids, where the addition of a cofactor during PrP amyloid formation can increase infectivity and pathogenicity of the prion, a recent study showed inclusion of the highly charged poly(adenosine 5′-diphosphate-ribose) (PAR) during fibrillization of α-synuclein allowed the generation of conformationally distinct PAR-α-synuclein fibrils that, when injected into the brains of mouse models, were 25-fold more neurotoxic than α-synuclein fibrils alone [82]. Recent near-atomic resolution cryo-electron microscopy structures of the tau filaments of Alzheimer’s disease, Pick’s disease, chronic traumatic encephalopathy (CTE), and corticobasal degeneration (CBD) indicate distinct structural tau conformers occur for each disease [83,84,85,86,87]. In addition, in the case of CTE and CBD tau filaments, additional densities were observed that the authors postulate may represent a non-proteinaceous cofactor. Much work remains to determine how different tau, Aβ, α-synuclein conformers, or strains correspond to clinical or pathological outcomes. However, the occurrence of aggregate “strains”, and the emerging implications of cofactor-dependent conformers for other proteopathies, in addition to the observance of misfolded proteins in the (seeming) absence of clinical signs in the human population, certainly suggests that misfolded proteins other than PrP prions may populate a spectrum of self-propagating states that may differ in their pathogenic outcomes.

## 6. Concluding Remarks

As the risks of clinically meaningful transmission of various proteopathies between humans are evaluated, the range of scenarios outlined above for PrP-based prion diseases, and the many experimental models thereof, must be considered. Clearly PrP-based prion diseases can represent deadly examples of transmissible proteopathies. However, an ability of a given ordered protein assembly to propagate in vitro, or even in vivo, does not necessarily mean that they are transmissible by any casual contacts or medical procedures, and, even if they are, that disease will result from that transmission. It seems likely that understanding of the real risks of transmission between humans will depend on careful epidemiological analysis. However, the execution and interpretation of such analyses can be complicated. For example, Collinge and colleagues recently reported evidence suggesting that, as has been well-documented in the transmission of prions of Creutzfeldt-Jakob disease (reviewed in [88]), amyloid-β pathology may have been transmitted to recipients of cadaveric growth hormone extracts containing amyloid-β aggregates [89]. However, an extensive earlier study by Trojanwski and colleagues failed to find evidence for the transmission of disease in such growth hormone recipients based on inoculation of proteins associated with Alzheimer’s disease, frontotemporal lobar degeneration-tau, or Parkinson’s disease (i.e., amyloid-β, tau, or α-synuclein) [90]. Nonetheless, given the much greater overall prevalence of these and numerous other proteopathies compared to Creutzfeldt-Jakob disease, it remains important to continue to assess the risks of even infrequent transmissions of prion-like protein aggregates that could either instigate or accelerate disease in humans.
